# Supplementary figures and images for: Molecular and electrophysiological features of spinocerebellar ataxia type seven in induced pluripotent stem cells
Source: PLoS One. 2021 Feb 24;16(2):e0247434. doi: 10.1371/journal.pone.0247434 (PMC7904216; doi:10.1371/journal.pone.0247434)

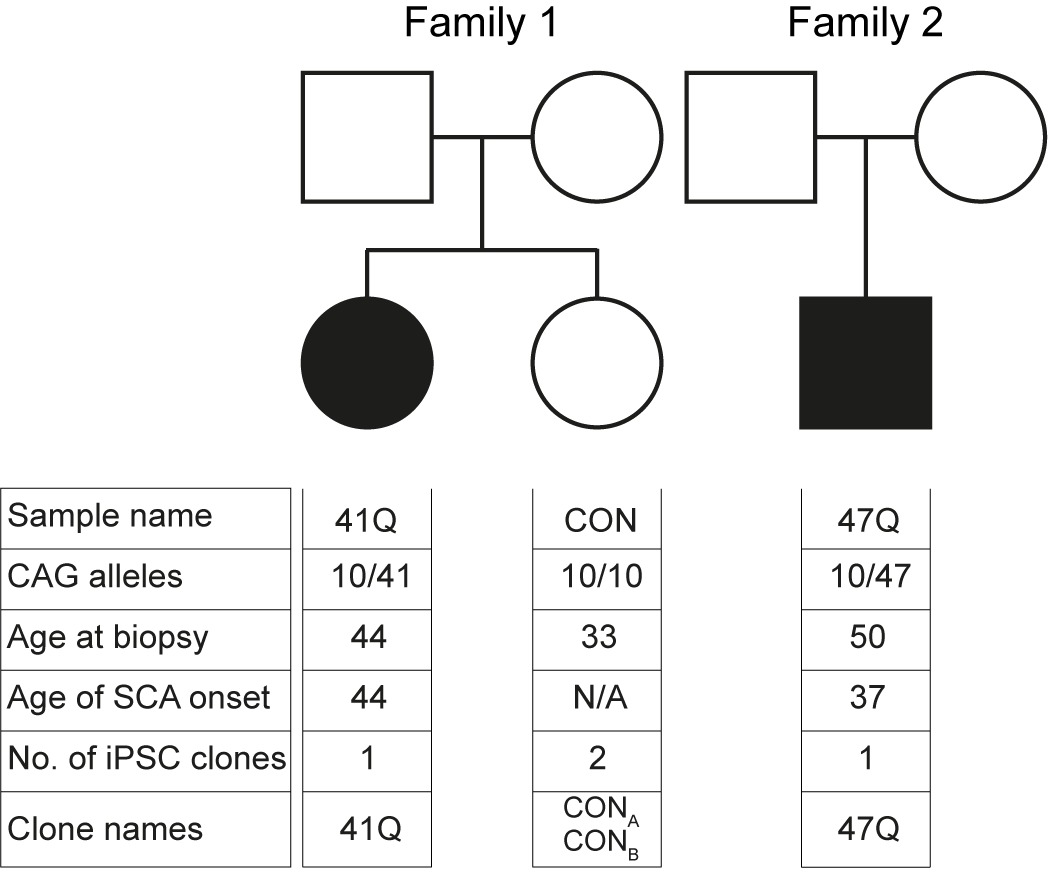

Supplement: S1 Fig — Genograms illustrating two families from which the SCA7 patient samples were taken from. A related control, sibling of 41Q, was used. From the three patients (two SCA7 and one control), 4 iPSC clones where derived (2 SCA7 lines and 2 Control lines). (TIF) [file pone.0247434.s001.tif]

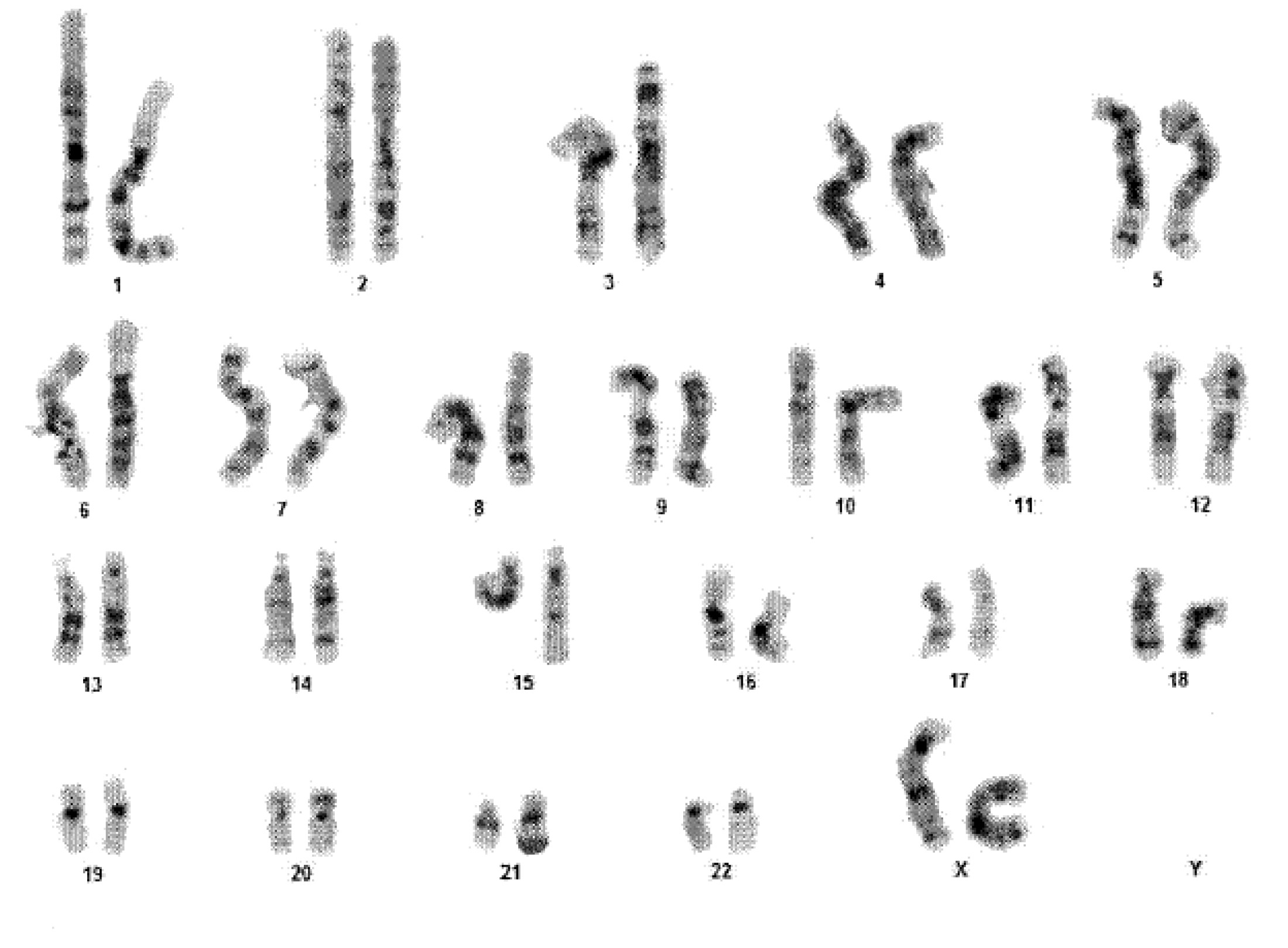

Supplement: S2 Fig — Karyogram from iPSC clone P1 showing no gross abnormalities. (TIF) [file pone.0247434.s002.tif]

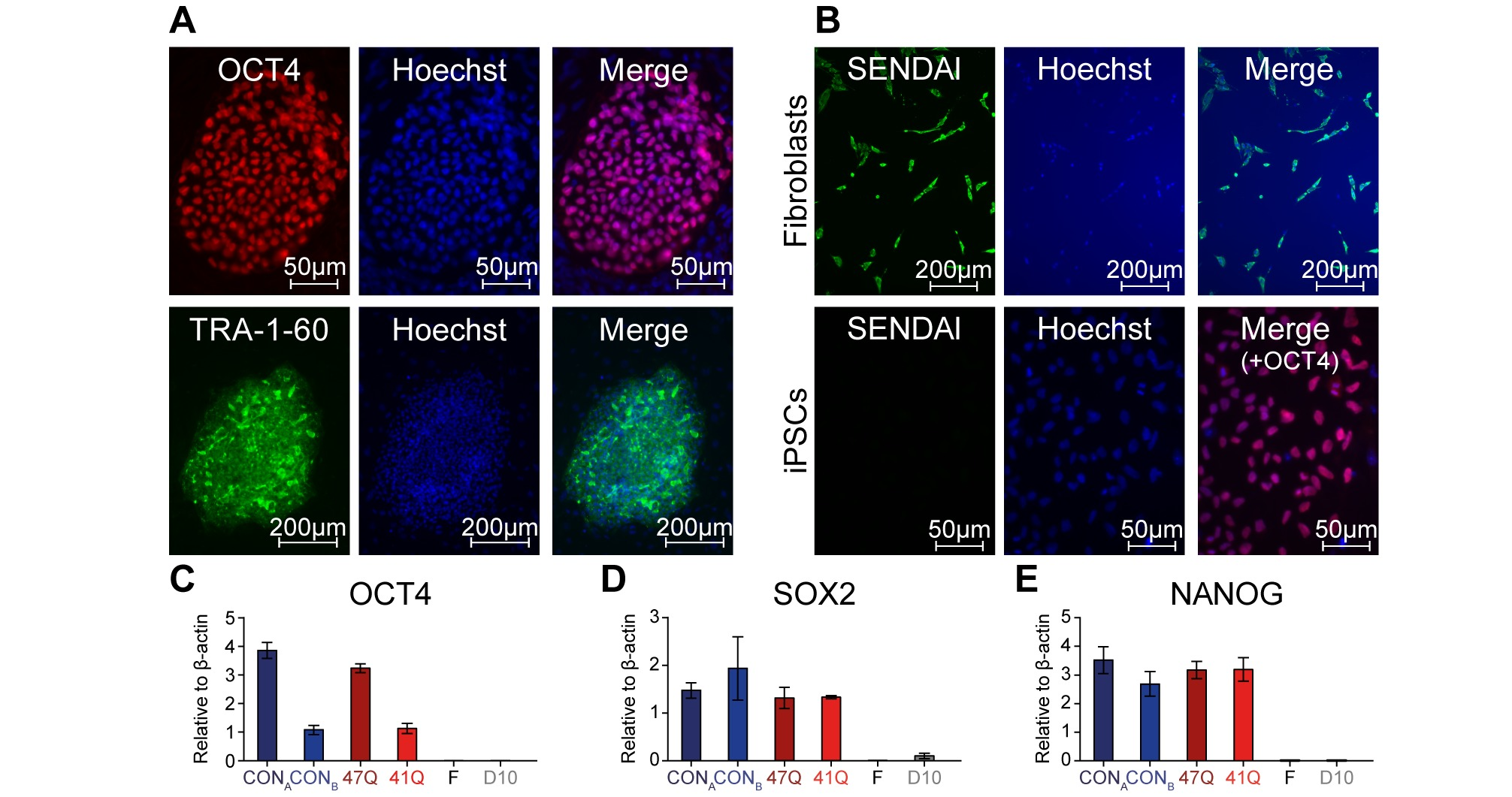

Supplement: S3 Fig — (A) Representative immunocytochemistry image showing positive OCT4 staining (red, top panel) and TRA-1-60 staining (green, bottom panel) in iPSCs clone 47Q. (B), Immunocytochemistry in newly infected fibroblasts (top panel) and iPSC colonies (bottom panel) co-stained with primary antibodies against OCT4 (red) and the viral nucleocapsid protein (green) showed effective silencing of the reprogramming Sendai virus. DAPI staining shown in blue. Expression of pluripotency markers in iPSC lines, determined by qRT-PCR. All five iPSC lines (47Q, 41Q, CONA, CONB) expressed OCT4 (C), SOX2 (D) and NANOG (E), compared to low expression levels in the original donor fibroblasts (F), or cells subjected to the retinal differentiation protocol for 10 days (D10, pooled data from lines 41Q and CONB). All levels shown relative to beta (β)-actin. (TIF) [file pone.0247434.s003.tif]

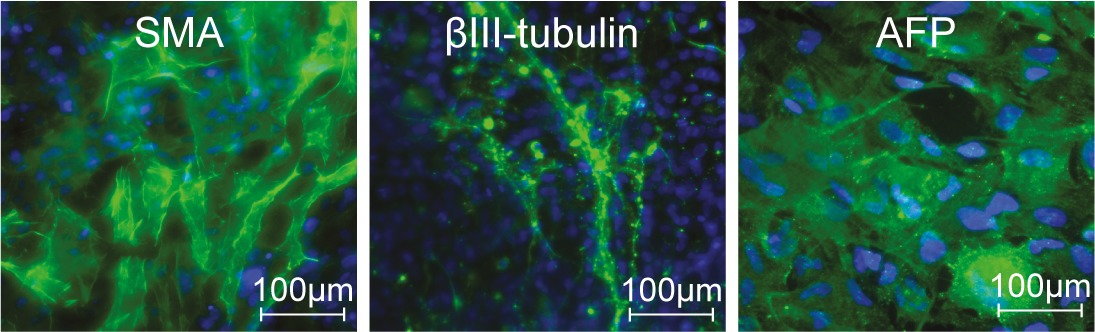

Supplement: S4 Fig — iPSCs were differentiated to three germ layers in vitro and validated by staining with appropriate markers: mesoderm using smooth muscle actin (SMA), ectoderm using β-III-tubulin, and endoderm using alpa-fetoprotein (AFP). Nuclei are counterstained with DAPI (blue). Images are representative of iPSCs from the 47Q patient clone. (TIF) [file pone.0247434.s004.tif]

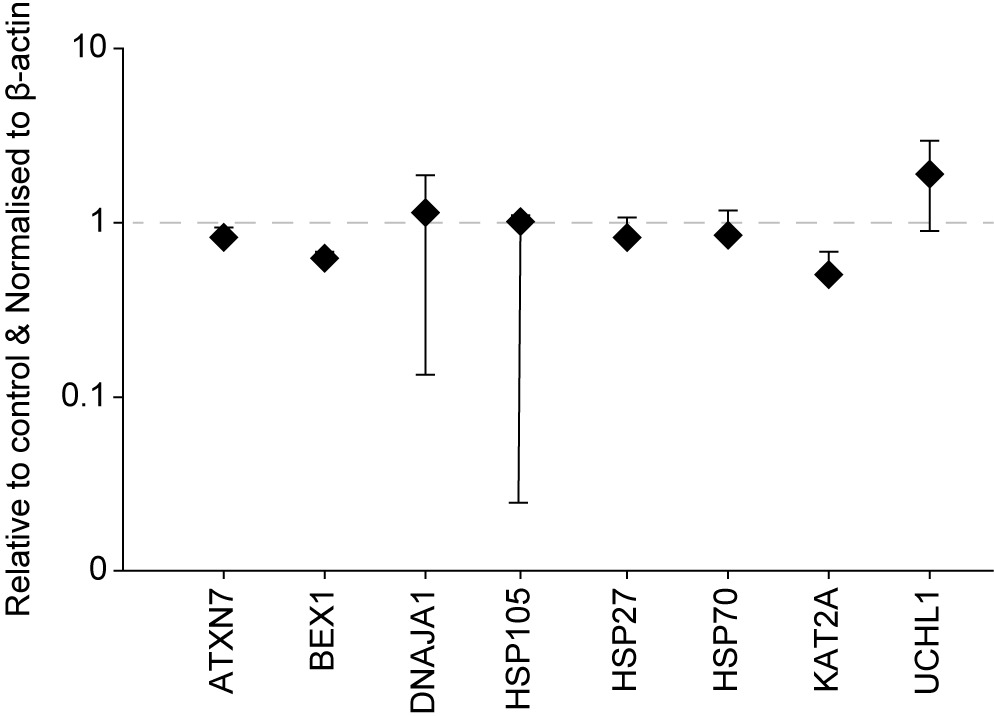

Supplement: S5 Fig — There were no significant transcriptional changes in SCA7 patient-derived fibroblasts compared to unaffected control fibroblasts. (TIF) [file pone.0247434.s005.tif]

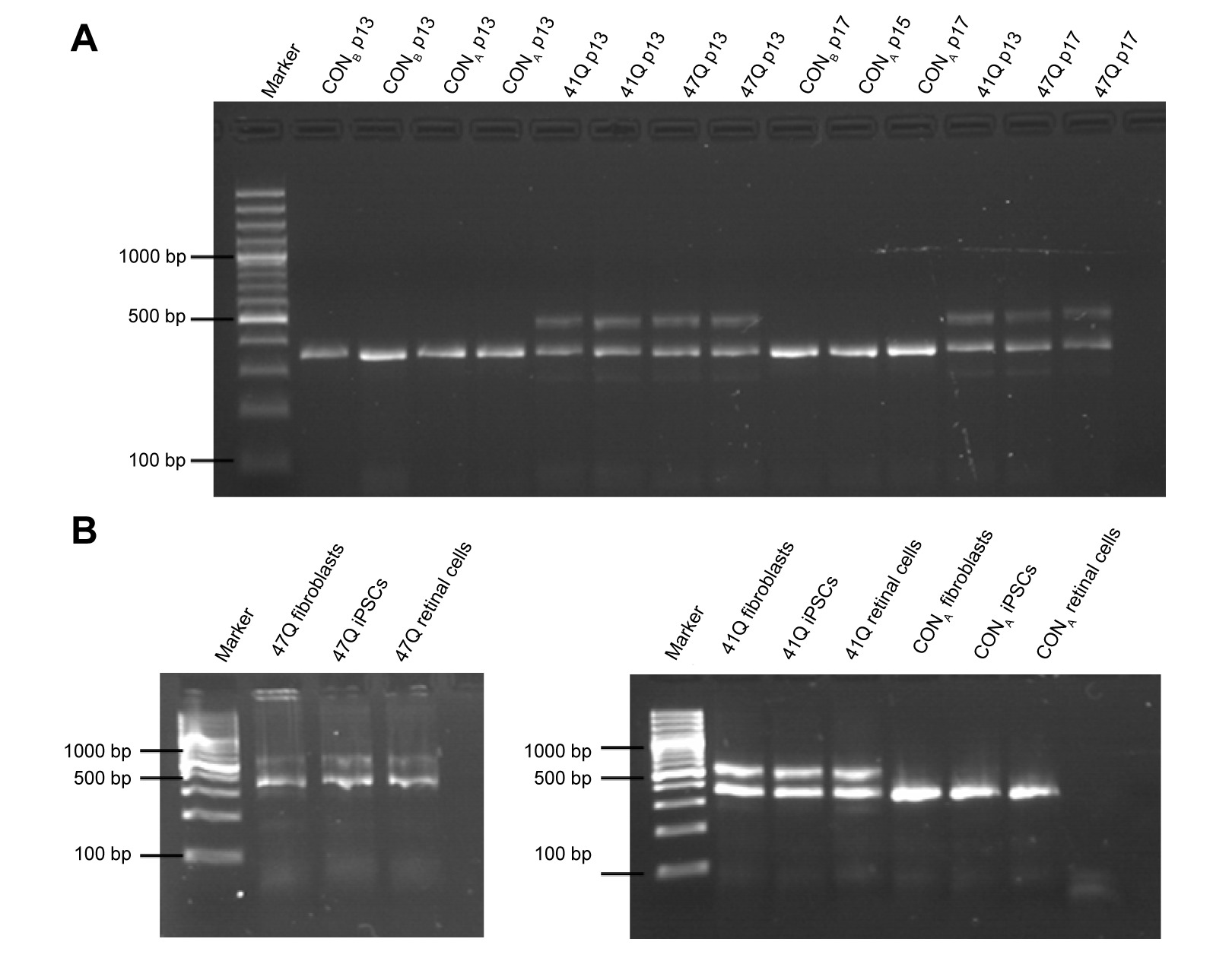

Supplement: S6 Fig — Gel electrophoresis after qRT-PCR on NPCs (A) and retinal cells (B). Samples from all three individuals showed a single band at approximately 355bp, corresponding with a wild-type allele, whilst patient cell lines 47Q and 41Q showed an additional larger band corresponding to a mutant allele. CAG repeat length in NPCs was evaluated at varying passages (indicated by p13, p15 or p17). (TIF) [file pone.0247434.s006.tif]
