# Supplementary material for: Molecular and electrophysiological features of spinocerebellar ataxia type seven in induced pluripotent stem cells
Source: PLoS One. 2021 Feb 24;16(2):e0247434. doi: 10.1371/journal.pone.0247434 (PMC7904216; doi:10.1371/journal.pone.0247434)
Supplement: S1 Raw images — (PDF) [file pone.0247434.s007.pdf]

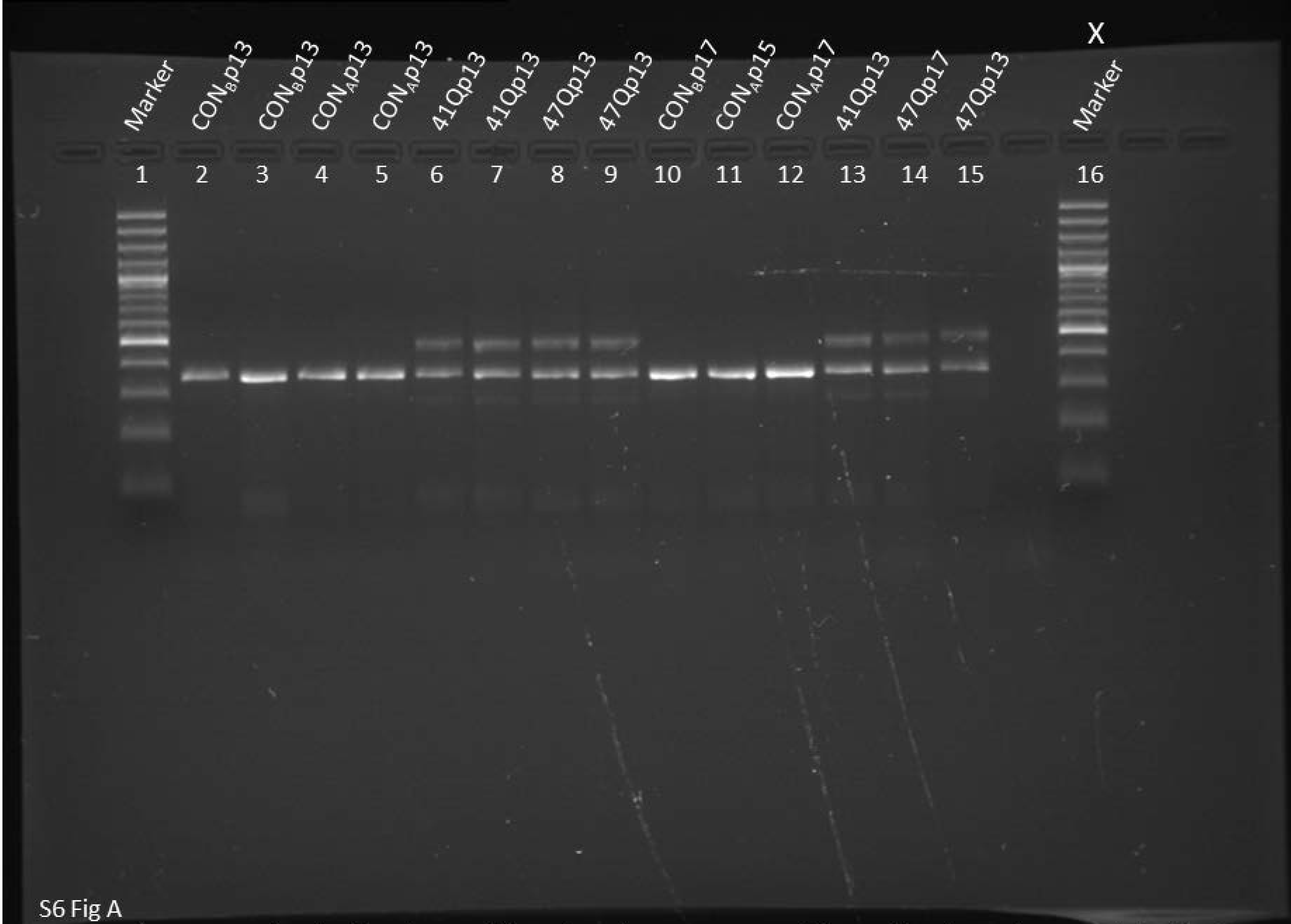

S6 Fig A

PCR products were visualised under UV light using the UVIPro UVIGold transilluminator (UVItec Limited). The UVIPro (version 12.3) software was used to capture and edit images.

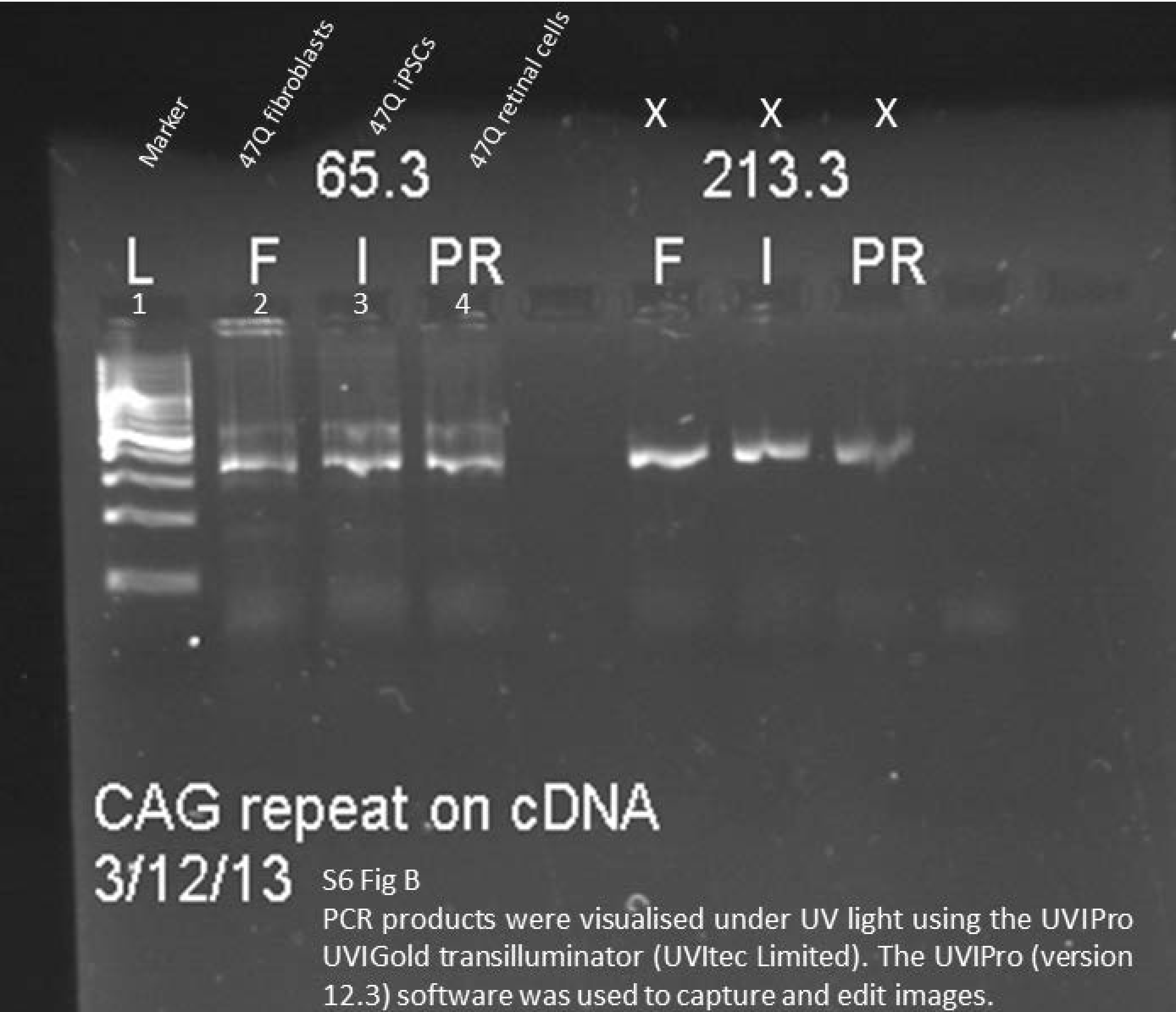

S6 Fig B

PCR products were visualised under UV light using the UVIPro UVIGold transilluminator (UVItec Limited). The UVIPro (version 12.3) software was used to capture and edit images.

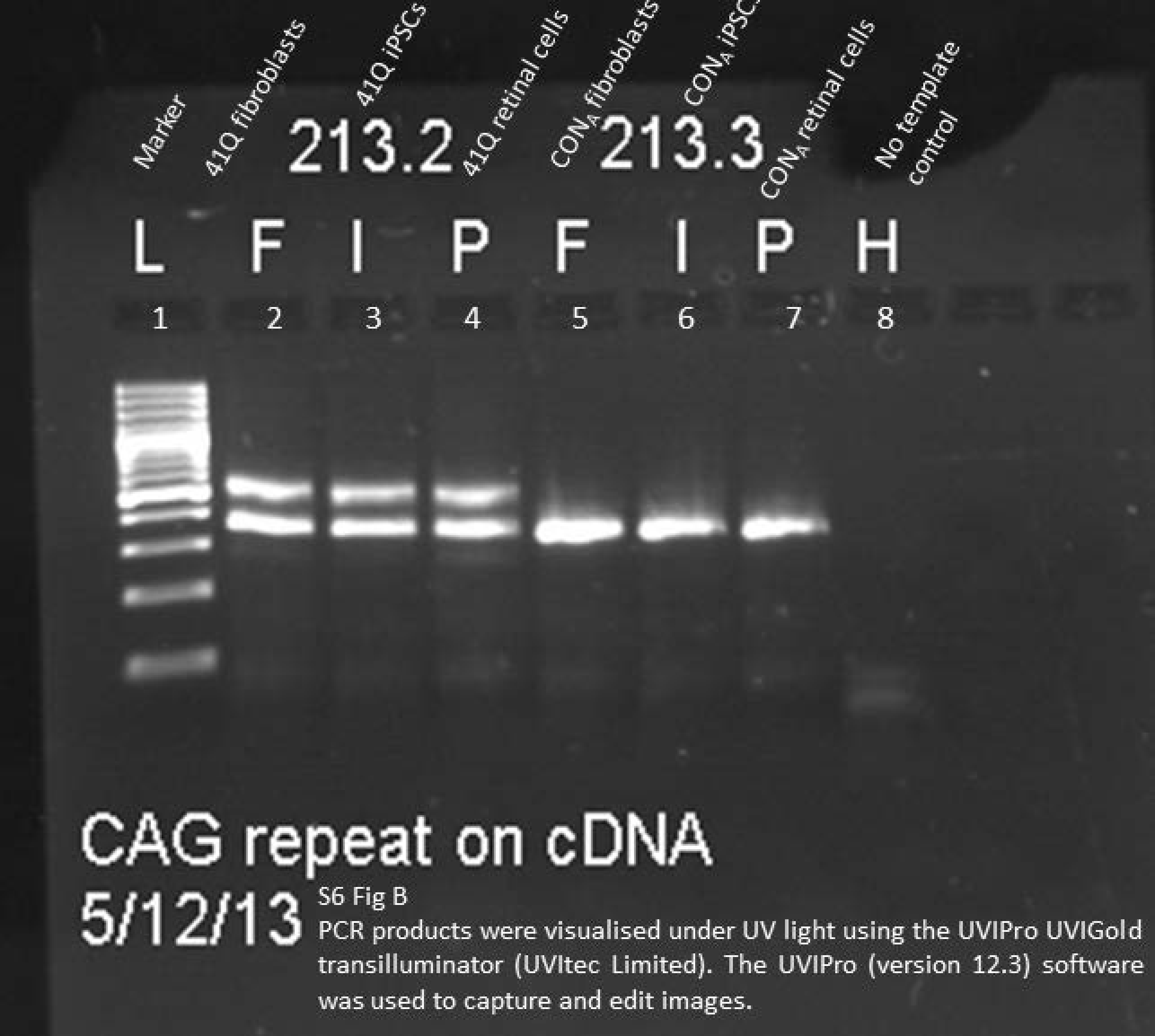

S6 Fig B

PCR products were visualised under UV light using the UVIPro UVIGold transilluminator (UVItec Limited). The UVIPro (version 12.3) software was used to capture and edit images.
