# Supplementary material for: Molecular and electrophysiological features of spinocerebellar ataxia type seven in induced pluripotent stem cells
Source: PLoS One. 2021 Feb 24;16(2):e0247434. doi: 10.1371/journal.pone.0247434 (PMC7904216; doi:10.1371/journal.pone.0247434)
Supplement: S1 Table — (DOCX) [file pone.0247434.s008.docx]

| **S1 Table: Primary antibodies** | | | | |
| --- | --- | --- | --- | --- |
| **Antibody** | **Full name** | **Species** | **Supplier and catalogue number** | **Dilution** |
| AFP | Alpha-fetoprotein | Mouse | Abcam, ab54745 | 1:100 |
| ASA | Anti-sarcomeric alpha actinin | Rabbit | Abcam, ab137346 | 1:100 |
| ATXN7 | Ataxin 7 | Rabbit | Thermo Scientific, PA1749 | 1:400 |
| CRX | Cone-rod homeobox | Sheep | R&D Systems, AF7085 | 1:20 |
| FOXA2 | Forkhead box A2 | Rabbit | Abcam, ab23630 | 1:1000 |
| GABA | γ-aminobutyric acid | Rabbit | Sigma-Aldrich,  A2052 | 1:500 |
| GFAP | Glial fibrillary acidic protein | Rabbit | Abcam, ab7260 | 1:100 |
| NES | Nestin | Mouse | Abcam, ab6320 | 1:1000 |
| NP | Nucleocapsid protein of Sendai virus | Mouse | Gift from Mahito Nakanishi | 1:1500 |
| OCT4 | POU class 5 homeobox 1 | Rabbit | R&D systems, KQX0409011 | 1:200 |
| RCVRN | Recoverin | Rabbit | Millipore, ab5585 | 1:1000 |
| SMA | Smooth muscle actin | Mouse | Abcam, ab7817 | 1:100 |
| TRA-1-60 (PODXL) Alexa-488 conjugated | Podocalyxin like | Mouse | Millipore, MAB4360A4 | 1:200 |
| βIII-Tubulin | Tubulin, beta 3 class III | Mouse | Abcam, ab78078 | 1:300 |
