# Supplementary material for: Molecular and electrophysiological features of spinocerebellar ataxia type seven in induced pluripotent stem cells
Source: PLoS One. 2021 Feb 24;16(2):e0247434. doi: 10.1371/journal.pone.0247434 (PMC7904216; doi:10.1371/journal.pone.0247434)
Supplement: S2 Table — (DOCX) [file pone.0247434.s009.docx]

| **S2 Table: Secondary antibodies** | | | | |
| --- | --- | --- | --- | --- |
| **Antibody** | **Dye** | **Species** | **Supplier** | **Dilution** |
| Anti-sheep | Alexa-488 | Donkey | Jackson Immunoresearch  **713-545-003** | 1:500 |
| Anti-rabbit | Cy3 | Donkey | Jackson Immunoresearch  **711-165-152** | 1:500 |
| Anti-mouse | Alexa-488 | Goat | Jackson Immunoresearch  **115-545-003** | 1:500 |
