# Supplementary material for: Molecular and electrophysiological features of spinocerebellar ataxia type seven in induced pluripotent stem cells
Source: PLoS One. 2021 Feb 24;16(2):e0247434. doi: 10.1371/journal.pone.0247434 (PMC7904216; doi:10.1371/journal.pone.0247434)
Supplement: S3 Table — (DOCX) [file pone.0247434.s010.docx]

| **S3 Table: Primer sequences** | | |
| --- | --- | --- |
| **Gene** | **Primer** | **Sequence (5' to 3')*** |
| ATXN7 | Atxn7 CAG RNA F | HEX-CGAGCTTTCAGAATGCAGC |
|  | Atxn7 CAG RNA F | CACTTCAGGACTGGGCAGAG |
| B-ACTIN | FORWARD | Proprietary Primer Design UK |
|  | REVERSE | Proprietary Primer Design UK |
| NANOG | FORWARD | CAGCCCCGATTCTTCCACCAG |
|  | REVERSE | CGGAAGATTCCCAGTCGGGTT |
| SOX2 | FORWARD | GGGAAATGGGAGGGGTGCAAA |
|  | REVERSE | TTGCGTGAGTGTGGATGGGAT |
| OCT3/4 | FORWARD | GACAGGGGGAGGGGAGGAGC |
|  | REVERSE | CTTCCCTCCAACCAGTTGCCC |
| MITF | FORWARD | TTCACGAGCGTCCTGTATGCAGAT |
|  | REVERSE | TTGCAAAGCAGGATCCATCAAGCC |
| NRL | FORWARD | GGTCCTAGTCCCAGCTCTTC |
|  | REVERSE | TCGTCCAATCCACATGAGAATTA |
| OTX2 | FORWARD | TGCAGGGGTTCTTCTGTGAT |
|  | REVERSE | AGGGTCAGAGCAATTGACCA |
| PAX6 | FORWARD | CGGAGTGAATCAGCTCGGTG |
|  | REVERSE | CCGCTTATACTGGGCTATTTTGC |
| RCVRN | FORWARD | CCAGAGCATCTACGCCAAGT |
|  | REVERSE | CACGTCGTAGAGGGAGAAGG |
| RHO | FORWARD | GTCGATTCCACACGAGCACTG |
|  | REVERSE | CCTCTCTGAATGGATACTTCGTC |
| RPE65 | FORWARD | GCCCTCCTGCACAAGTTTGACTTT |
|  | REVERSE | AGTTGGTCTCTGTGCAAGCGTAGT |
| ARR3 | FORWARD | TCACTTCCAAGTCATCACGG |
|  | REVERSE | GGTGTTGTCCTGGTTGATCC |
| GNAT1 | FORWARD | TAGCTGAGGGGAGTGCAAAT |
|  | REVERSE | CCTCAAAGACTGTGGCCTCT |
| ATXN7 | FORWARD | GCCAGCCGTGAACAATGTC |
|  | REVERSE | TTCCTCCCCGTGCTATTTTCA |
| BEX1 | FORWARD | GGAGGAGACTACAAGGATAGG |
|  | REVERSE | TCCTTTTCTTCATTTTCTTGGTT |
| DNAJA1 | FORWARD | AAAGGAGGAGAACAGGCAATTAA |
|  | REVERSE | TAGGGTTACTGAGAGCTGATGT |
| GRIA2 | FORWARD | CTATGGCATCGCAACACCTAA |
|  | REVERSE | GTCCTTGGCTCCACATTCAC |
| HSP27 | FORWARD | ACGAGCTGACGGTCAAGAC |
|  | REVERSE | GGGGGCAGCGTGTATTTCC |
| HSP70 | FORWARD | ATGGAATCTATAAGCAGGATCT |
|  | REVERSE | CACATACAGAAACTTGATAAGC |
| HSP105 | FORWARD | CCCGTCAGTCATATCATTTGGA |
|  | REVERSE | AATCTTTTGAAGTTAGACACCGTATT |
| OLIG1 | FORWARD | GTTTGGAGAGCTGTATTTAAGACT |
|  | REVERSE | TTCTAAGAAACCCCCAGGATTTA |
| UCHL1 | FORWARD | TGAAGCAGACCATTGGGAAT |
|  | REVERSE | TGTTTCAGAACTGATCCATCCT |
| PLCB3 | FORWARD | CCTTGGAAATCTTTGAGCGGTTC |
|  | REVERSE | ACTTCGTTGAGTCTCGGGTC |
