# Supplementary material for: Molecular and electrophysiological features of spinocerebellar ataxia type seven in induced pluripotent stem cells
Source: PLoS One. 2021 Feb 24;16(2):e0247434. doi: 10.1371/journal.pone.0247434 (PMC7904216; doi:10.1371/journal.pone.0247434)
Supplement: Attachment — Submitted filename: burman et al Response to Reviewers Jan 2021.docx [file pone.0247434.s011.docx]

Jan 2021

We thank the Editor and Reviewers for their thoughtful comments and suggestions. In the rebuttal below, we have addressed all the points with our responses shown in *italics*. We have also highlighted all changes to the original manuscript in yellow.

**Reviewer #2:**

Reviewer: The majority of my comments was sufficiently addressed, except for the comment concerning interpretation of the results based on two SCA7 cell lines analyzed.
First of all, I am not convinced by explanation for not generating isogenic cell lines by comparison with DMPK gene. I know that the CRISPR/Cas-9-based strategy is difficult to be applied for repeated tracts and its application for this study would take a long time. Still, there are studies like PMID: 28238795 and 32182692 which show application of such strategy for huntingtin, where CAG repeat tract is also present in ORF. It can be referred and commented instead.

*Response:* *We thank the reviewer for these comments and agree with what has been suggested. We have revised the manuscript to expand our discussion on future experiments and the application of CRISPR/Cas-9-based strategies (see lines 548-557). We also thank the Reviewer for the references they have suggested which we have now included in our manuscript.*

Reviewer: Unfortunately, in attached files I could not access current version of Fig. 3. (zip files contained only main figures 1 and 4) which was changed as I see in this figure legend and the interpretation.

*Response: We apologise to the Reviewer for this technical glitch. We can confirm that all the Figures were successfully uploaded during the resubmission. We have ensured that all Figures are again uploaded successfully upon submission of this revision.*

**Reviewer #5:**

Reviewer: Unfortunately the revised manuscript "Molecular and electrophysiological features of spinocerebellar ataxia type seven in induced pluripotent stem cells" does not lead to any improvement in either of the two main critical points:

1.) Since the line to line variability is too high, no conclusive results can be obtained.
2.) Even more important, the identity of differentiated cell types (either neurons or retinal photoreceptors) is still more or less unknown. As the authors stated in their response: "The low cell numbers and heterogeneity of mature neuronal cultures
proved a challenge to obtaining significant biological material to perform reliable expression analysis." If even such an analysis was not possible, then the results of the electrophysiological recordings are inappropriate. The maturity and the cellular composition/identity of differentiated neurons determines the electrophysiological properties. Therefore, it cannot be excluded that the "features of functional aberrations" in SCA7 iPSC-derived neurons are a cell culture artefact rather than a consequence of the mutation. Without any additional experiments to prove the identity of differentiated neurons (proportion of GABAergic vs. glutamatergic neurons / neuronal subtype specification) and photoreceptors no conclusive findings can be formulated.

*Response: We thank the Reviewer for their critical appraisal of our study. We agree with the Reviewer that given the significant variability between cell lines, it is not appropriate to make conclusive statements from the findings. We have, however, avoided from doing so and have instead aimed to present our results as they are whilst fully acknowledging the limitations. In this revision, we have ensured that the tone of our manuscript does not mislead the reader into thinking that our findings are conclusive and have again highlighted the limitations. In addition, we clearly highlight what future research is needed to take the work forward.*

*We do believe our paper does still provide technical insights that may prove useful to others especially those considering embarking on developing or refining iPSC models of SCA7. While we agree that the experiments you suggest to prove the identity of the differentiated cell are warranted, at present this is not possible. This is largely due to the significant negative impact the ongoing COVID-19 pandemic has had on our research efforts as well as the ongoing restrictions it is having on our capacity.*
